# The Lifestyle Intervention in memory clinics of General and academic Hospitals Trial (LIGHT): Rationale and study design of a randomized controlled trial to reduce modifiable dementia risk

**DOI:** 10.1186/s13195-025-01947-9

**Published:** 2026-01-08

**Authors:** V. van Gils, L. Waterink, S. C. P. M. Wimmers, J. G. M. Jelsma, M. E. de Vugt, R. Handels, S. A. M. Sikkes, W. M. van der Flier, K. Deckers, M. D. Zwan, S. Köhler, N. Janssen

**Affiliations:** 1 Department of Psychiatry and Neuropsychology, Alzheimer Centrum Limburg, Mental Health and Neuroscience Research Institute (MHeNs), Maastricht University, Maastricht, The Netherlands; 2 Alzheimer Center Amsterdam, Neurology, Vrije Universiteit Amsterdam, Amsterdam UMC Location VUmc, Amsterdam, The Netherlands; 3 Amsterdam Neuroscience, Neurodegeneration, Amsterdam, The Netherlands; 4 Precision Health Clinic, Amsterdam, The Netherlands; 5 Department of Public and Occupational Health, Amsterdam UMC, location Vrije Universiteit Amsterdam, Amsterdam, The Netherlands; 6 Amsterdam Public Health research Institute, Health Behaviors & Chronic Diseases, Amsterdam, The Netherlands; 7 Amsterdam Public Health research Institute, Quality of Care, Amsterdam, The Netherlands; 8 Division of Neurogeriatrics, Department of Neurobiology, Care Sciences and Society, Karolinska Institutet, Solna, 171 64 Sweden; 9 Faculty of Behavioural and Movement Sciences, Department of Clinical, Neuro and Developmental Psychology, Vrije Universiteit, Amsterdam, The Netherlands; 10 Alzheimer Nederland, Amersfoort, The Netherlands; 11 Department of Epidemiology and Data Science, Amsterdam University Medical Center, Vrije Universiteit Amsterdam, Amsterdam, The Netherlands; 12 Amsterdam Public Health, Amsterdam, The Netherlands

**Keywords:** Lifestyle, Intervention, LIfestyle for brain health (LIBRA), Dementia, Risk reduction, Memory clinic, Cognitive function

## Abstract

**Introduction:**

Dementia risk reduction through lifestyle modification has much potential but is not yet implemented in routine clinical care. Currently, there are no preventive interventions available for memory clinic patients. Therefore, the aim of The Lifestyle Intervention in the memory clinics of General and academic Hospitals Trial (LIGHT) is to examine the (cost)effectiveness of a multidomain intervention combining lifestyle coaching with risk self-management for patients with subjective cognitive decline (SCD) and mild cognitive impairment (MCI).

**Methods:**

LIGHT is a 1-year multi-center randomized controlled trial for dementia risk reduction by improving brain health through lifestyle modifications in memory clinic patients without dementia. Starting early 2025, the trial aims to include 300 older adults (≥ 50 years) with SCD or MCI, with presence of ≥ 2 modifiable dementia risk factors, recruited via the memory clinics of Dutch hospitals. Participants are randomized 1:1 to either the intervention group or control group. The intervention consists of three components: (1) three individual sessions with a certified lifestyle coach to set and work on personal goals, (2) vouchers for access to brain-healthy services from local providers, and (3) access to an online self-management platform (www.breinzorg.nl) providing psychoeducation on dementia risk reduction through lifestyle. The control group receives general health advice. The primary outcome is 1-year change in modifiable dementia risk captured by the LIfestyle for BRAin Health 2 (LIBRA2) index consisting of coronary heart disease, diabetes, hypercholesterolemia, hypertension, depression, obesity, smoking, high physical activity, and chronic kidney disease, high alcohol intake, high cognitive activity, healthy diet, hearing impairment, sleep disturbances, and social participation. Secondary outcomes include cognitive functioning, health-related quality of life, activities of daily living, self-efficacy, care use, as well as change in individual risk factors.

**Conclusion:**

LIGHT will provide insight into the implementation and (cost-)effectiveness of a lifestyle intervention for indicated prevention in a memory clinic setting.

**Trial registration:**

Clinicaltrials.gov: NCT06832761 (date 2025-02-18), OMON: 57,198.

**Supplementary Information:**

The online version contains supplementary material available at 10.1186/s13195-025-01947-9.

## Background

Dementia is a major public health challenge according to the World Health Organisation [1]. Currently, over 57 million people live with dementia worldwide, increasing to over 150 million cases in 2050 [2]. Over the past decade, increasing evidence suggests that universal (targeting entire populations) or selective (targeting specific groups within populations) prevention of dementia through lifestyle modifications could have large impact [3]. Dementia has a multifactorial aetiology, for which modifiable risk factors jointly account for about 45% of dementia cases worldwide [3, 4]. Most of these factors relate to cardiometabolic health, such as hypertension, hypercholesterolemia, obesity, diabetes, low physical activity, and smoking, but other lifestyle factors such as depression, poor sleep, lack of social contacts, or low cognitive activity also contribute.

Occurrence of risk factors vary widely across individuals, and effective risk reduction requires personalized and multidomain lifestyle interventions [5, 6]. Some previous lifestyle intervention studies found improvement or reduced decline in cognition following the intervention [7–9], but most studies generally did not find an effect of multidomain lifestyle interventions on cognitive outcomes [6, 10–12]. This might be due to problems with adherence to the intervention and relatively short follow-up periods [13]. However, when considering brain health or dementia risk scores such as LIfestyle for BRAin Health (LIBRA), ANU Alzheimer’s Disease Risk Index (ANU-ADRI), or Cardiovascular Risk Factors, Aging, and Incidence of Dementia (CAIDE), studies consistently show decreased dementia risk scores across the multidomain lifestyle intervention groups [7, 11, 14–16]. These results are promising and suggest that implementing multidomain lifestyle interventions may help change people’s behavior towards a healthier lifestyle to effectively reduce dementia risk.

So far, most research comes from studies in the general population, while evidence for indicated prevention in memory clinics (in stages of subjective cognitive decline (SCD) and mild cognitive impairment (MCI)), is lagging behind. These patients generally have worse health and lifestyle profiles and are at higher risk for dementia [17, 18]. While they might, thus, benefit from preventive measures, there are currently no integrated preventive measures for dementia risk reduction in routine clinical care [19]. Real-world implementation of interventions studied in earlier WW-FINGER trials is difficult due to strict programs and high intensity [20]. Therefore, it is necessary to explore more personalised intervention options and to assess their implementability in healthcare.

Therefore, the primary objectives of the Lifestyle Intervention at General and academic Hospitals Trial (LIGHT) are to: (1) test the effectiveness of a lifestyle intervention on improving the dementia risk profile of people with SCD and MCI in a randomized controlled trial (RCT), as compared to general health advice (2) identify barriers, facilitators and evaluate strategies for sustainable implementation of the lifestyle intervention, and (3) estimate the cost-effectiveness of the lifestyle intervention from a lifetime horizon and societal perspective.

## Methods

### Study design

LIGHT is a multi-center RCT assessing the (cost-)effectiveness of a 1-year personalized lifestyle intervention as compared to usual healthcare and general health advice with 300 participants. Participating research centers include Maastricht University and Amsterdam University Medical Center. Outcome measurements are conducted at baseline and at the end of the 1-year intervention. LIGHT has been approved by the medical ethical committee of MUMC+ (NL86513.068.24) and is registered at Dutch trial register OMON (NL-OMON57198) and ClinicalTrials.gov (NCT06832761, registration date February 18th 2025). This study will be conducted in accordance with the ethical conduct and juridical laws of the Declaration of Helsinki (2013), Good Clinical Practice (GCP) as defined by the International Conference on Harmonization (ICH) Guidelines, and the Medical Research Involving Human Subjects Act (WMO). Results will be reported in line with the Consolidated Standards Of Reporting Trials (CONSORT) guidelines [21].

### Inclusion criteria

Inclusion criteria are: 1) ≥ 50 years of age at pre-screening; 2) memory clinic patients with clinician-confirmed SCD or MCI; 3) Presence of ≥ 2 modifiable risk factors for dementia specified by the updated LIBRA2 score [22, 23]. Persons were classified as SCD when being referred to the memory clinic with subjective cognitive complaints but without showing objective cognitive impairments on neuropsychological assessment [24]. A diagnosis of MCI was defined by impairment (<−1.5 SD below normative mean) on at least one cognitive domain measured by neuropsychological assessment [25]. Only risk factors that are potentially modifiable within one year are considered f or inclusion (i.e., hypercholesterolemia, hypertension, depressive mood, obesity or overweight, current smoking, low physical activity, high alcohol consumption, low cognitive activity, unhealthy diet, poor sleep quality, low social participation).

### Exclusion criteria

Exclusion criteria are: (1) having a clinician-confirmed diagnosis of dementia as defined by the DSM-V criteria; (2) insufficient understanding of the Dutch language; (3) conditions affecting safe and continuous engagement in the intervention (e.g. under treatment for current malignant diseases, major psychiatric disorders (e.g. major depression, psychosis, bipolar disorder), other conditions preventing co-operation as judged by the local study nurse or consulted physician at the local study site; (4) participation in any other research intervention trial at time of pre-screening and throughout the study period.

### Recruitment and pre-screening

Recruitment takes place in memory clinics of academic (Maastricht University Medical Center + and Amsterdam University Medical Center) and general hospitals in the Netherlands. Patients are actively recruited by physicians and psychologists at the participating memory clinics through explanation, flyers, posters, etc. Both memory clinics of the participating centers have an established research infrastructure, increasing feasibility of recruitment. Other recruitment routes involve large ongoing cohort studies at the memory clinics. Potentially eligible participants first receive detailed oral explanation by the researchers, and, if interested, this is followed up by written information about the study. After receiving the information, participants will have at least 7 days before deciding on participation. The researcher then performs a pre-screening to check eligibility based on the inclusion criteria through a short telephone questionnaire. Eligible participants are invited for the baseline visit, where written informed consent is signed.

### Randomization and blinding

After the baseline visit, participants are randomized with a 1:1 ratio to either the intervention group or control group. Block randomization (in variable groups of two and four) stratified for study centre and diagnosis (SCD or MCI) is used. Randomization is done by a computer-generated allocation scheme integrated in the Castor electronic data capturing system (Castor EDC, Amsterdam, The Netherlands). Researchers executing cognitive assessments at follow-up are blinded for group allocation. Group allocation is not actively disclosed to participants, but complete blinding is unlikely due to the nature of the intervention.

### Personalized lifestyle intervention

**Fig. 1:**
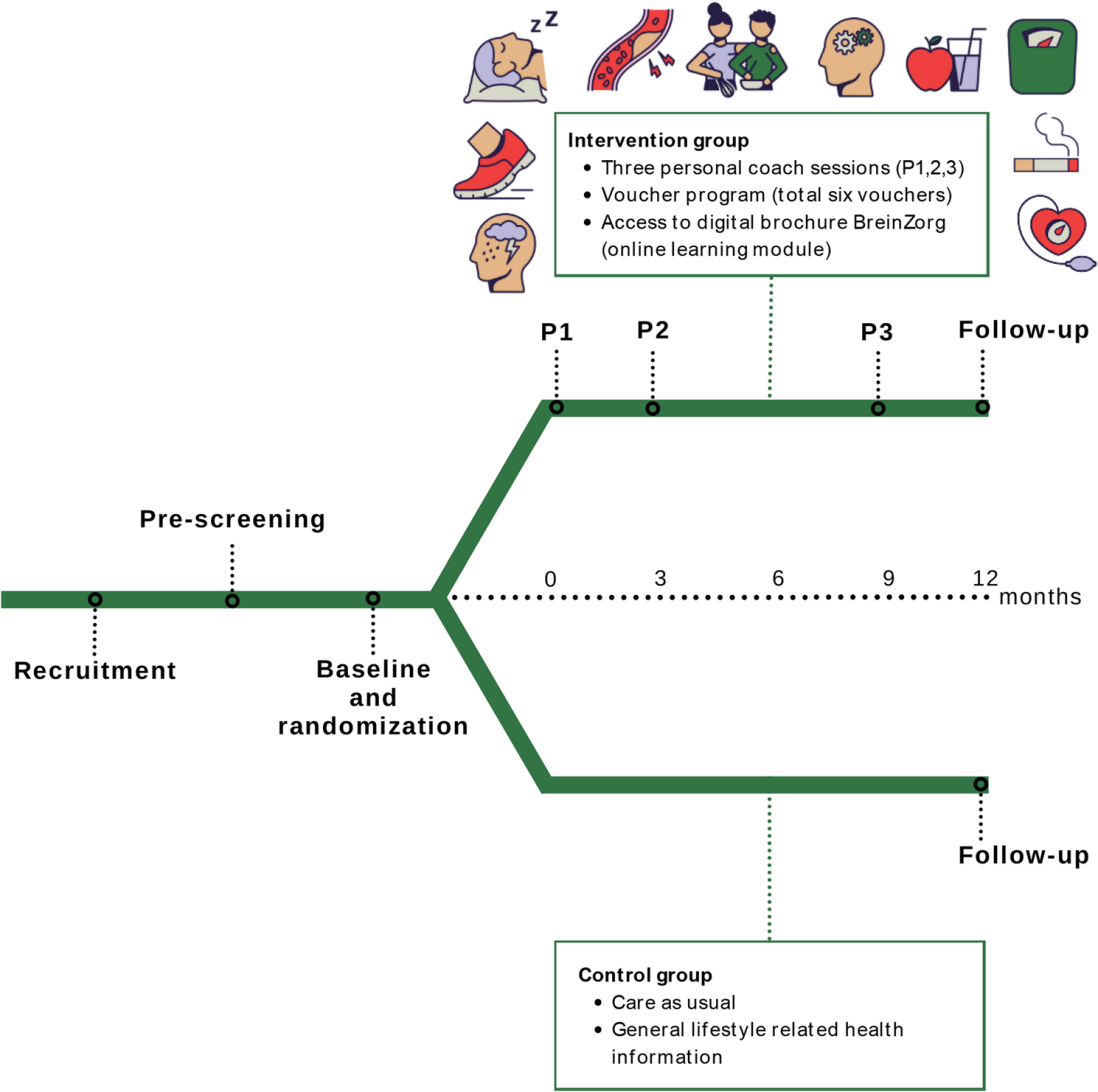
LIGHT study design

An overview of the study design is shown in Fig. 1. The personalized lifestyle intervention includes three components:

1. Lifestyle coach

The first and main component of the intervention involves three personal sessions with a certified lifestyle coach. At the start of the intervention (2–4 weeks after the baseline assessment), the coach will provide tailored advice (on site or through an online video call). The coach discusses the individual lifestyle risk profile (based on baseline LIBRA2 measures, see below) and through motivational interviewing [26], suggests lifestyle changes based on the risk factors with suggested room for improvement. The participant and lifestyle coach agree on an individual plan and set realistic personal goals, also considering the personal aims and wishes of the participant. Tailored advice may for example comprise specific advice on how to be more physically active or to adopt more healthy dietary choices based on the participants’ personal preference and motivation. Participants may be referred to suitable professional help (e.g. dietician, stop smoking program) or community-based lifestyle initiatives (e.g. free sports initiatives for elderly, cooking groups) to facilitate and maintain behavioural change. Two follow-up sessions 3 and 9 months after the first visit are intended to discuss progress and, if needed, adjust or set new personal goals. Sessions will take 1 to 1.5 h.

2. Voucher program

The second component is a voucher program, which was co-developed with regional partners, to support participants in making a first step towards a brain-healthy lifestyle. The voucher program is inspired by a similar program that is implemented in the Programme Dementia Prevention (pdp) in Luxembourg [27]. Participants receive up to two vouchers from the lifestyle coach per visit (with a total of six vouchers, each worth 50 euros) to support brain healthy behaviours tailored to the individual risk profile and goals as discussed with the lifestyle coach. These vouchers provide access to services across multiple domains: physical activity, social participation, cognitive activity, and healthy diet and include, among others, gym memberships, free entrance to museums, or fresh vegetable and fruit from local farmers. The lifestyle coach has an advisory and motivational role in the voucher usage.

3. Access to online platform

Finally, participants receive access to the online risk self-management platform BreinZorg (‘BrainCare”, www.breinzorg.nl). The platform was developed through co-creation with the target group and provides psychoeducation, tips, and exercises to maintain brain health, with learning modules for each LIBRA2 factor. Participants will also receive a binder with general information about the LIBRA2 factors in relation to brain health and dementia. It additionally includes the participants’ personal risk profile, personal goals and evaluation, and instructions for the usage of the vouchers and BreinZorg.

### Control group

The control group receives general health advice through a similar binder, including the same general information about the LIBRA2 factors in relation to brain health and dementia. Participants in both groups will receive regular online newsletters and updates as well as gift vouchers (75 euros in total) for their participation.

### Baseline and outcome measurement

All study participants undergo a baseline measurement and one follow-up outcome measurement at 12 months at the research site. Measurements include an assessment of demographics, medical history and medication, cognitive testing, clinical measures, blood sampling and questionnaires. In total, research visits take up to 1.5 h. Table 1 gives an overview of all primary and secondary outcomes.

**Table 1:** LIGHT data collection

Measures	Type of outcome
**Lifestyle score**	
LIfestyle for BRAin Health 2 (LIBRA2)	Primary
**Cognition**	
15-Word Verbal Learning Test	Secondary
Digit Symbol Substitution Test 90 s	Secondary
Wechsler Adult Intelligent Scale (WAIS) digit span	Secondary
Trail Making Test (TMT)	Secondary
Semantic Fluency	Secondary
**Blood-based markers**	
Cholesterol levels (total, HDL, LDL, triglycerides)	Secondary^1^
HbA1c levels, %	Secondary^1^
Creatinine-based eGFR levels	Secondary^1^
**Clinical measures**	
Height and weight (BMI)	Secondary^1^
Blood pressure (systolic and diastolic)	Secondary^1^
**Medical history and medication**	
Diabetes (and type)	Secondary^1^
Coronary heart disease	Secondary^1^
Kidney disease	Secondary^1^
Hypertension	Secondary^1^
Hypercholesterolemia	Secondary^1^
Depression	Secondary^1^
Hearing loss	Secondary^1^
Sleep disorders	Secondary^1^
Smoking (quantity, duration)	Secondary^1^
Alcohol intake (quantity, duration)	Secondary^1^
**Questionnaires**	
* Lifestyle*	
Mediterranean Diet Adherence Screener (MEDAS)	Secondary^1^
European Prospective Investigation into Cancer and Nutrition (EPIC) physical activity questionnaire	Secondary^1^
Cognitive and Leisure Activities Scale	Secondary^1^
Perceived Stress Scale	Secondary
Patient Health Questionnaire (PHQ)−9	Secondary^1^
Groningen Sleep Quality Scale (GSKS)	Secondary^1^
6-item Lubben social network Scale	Secondary^1^
De Jong Gierveld 6-item Scale	Secondary
* Daily functioning and psychological*	
Amsterdam-Instrumental Activities of Daily Living (ADL)-Questionnaire	Secondary
Health locus of Control Scale	Secondary
General Self-Efficacy scale	Secondary
Pearlin Mastery Scale	Secondary
* Knowledge*	
Knowledge on dementia risk questionnaire	Secondary
**Process evaluation**	
Mixed-method on implementation, context, mechanism of impact (questionnaires, interviews, focus-groups, data registration)	Secondary
**Cost-effectiveness**	
EuroQol-5D-5 L	Secondary
ICEpop CAPability measure for Older people (ICECAP-O)	Secondary
Medical Consumption Questionnaire (iMCQ)	Secondary
Productivity Costs Questionnaire (iPCQ)	Secondary

All measures were performed at baseline and 12 months

*Abbreviations*: *LIBRA2 *LIfestyle for BRAin health 2, *WAIS *Wechsler Adult Intelligence Scale, *TMT *Trail Making Test, *HDL *high-density lipoprotein, *LDL *low-density lipoprotein, *HbA1c* glycated hemoglobin, *eGFR *estimated Glomerular Filtration Rate, *BMI *body mass index, *MEDAS *Mediterranean Diet Adherence Screener, *EPIC *European Prospective Investigation into Cancer and Nutrition, *PHQ-9 *Patient Health Questionnaire-9, *GSKS *Groningen Sleep Quality Scale, *ADL *Activities of Daily Living, *ICECAP-O* ICEpop CAPability measure for Older people, *MCQ *Medical Consumption Questionnaire, *PCQ *Productivity Costs Questionnaire

^1^Primary outcome as part of LIBRA2, secondary outcome as assessed individually

### Primary outcome

**Fig. 2:**
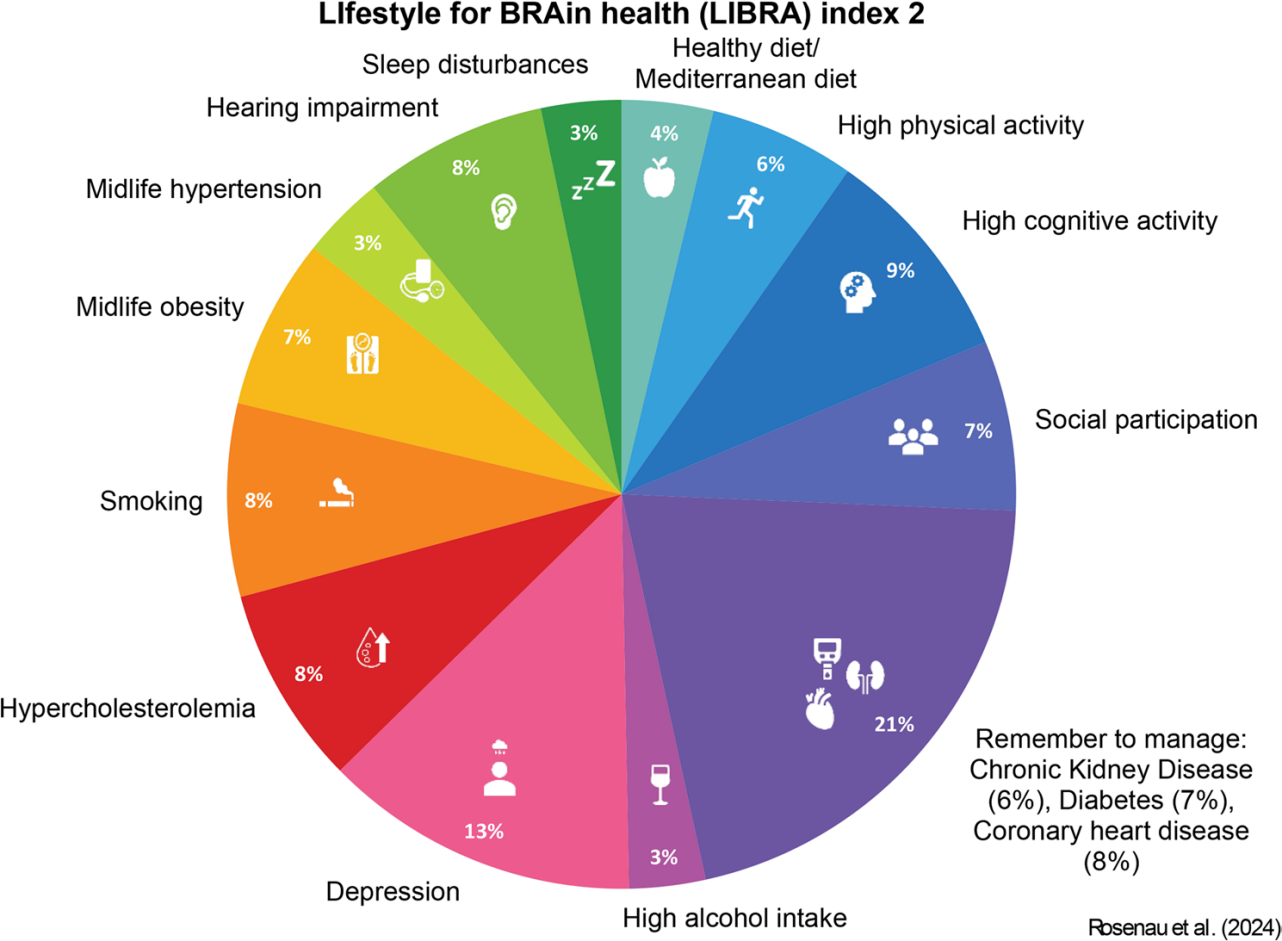
Risk and protective factors captured by the Lifestyle for Brain Health score (LIBRA2). Percentages indicate the weight of the risk factors involved as calculated by LIBRA2

The primary outcome is the 1-year change in participants’ modifiable dementia risk profile as measured by the LIBRA2 score (Fig. 2). LIBRA2 comprises of 15 modifiable dementia risk and protective factors identified through an umbrella review and expert consensus study: coronary heart disease, diabetes, hypercholesterolemia, hypertension, depression, obesity, smoking, high physical activity, chronic kidney disease, high alcohol intake, high cognitive activity, healthy diet, hearing impairment, sleep disturbances, and social participation [4, 22]. Each of these factors has an assigned weight based on the factor’s relative risk for dementia from meta-analyses as shown in Supplementary Table 1 [23]. Based on the presence or absence of risk or protective factors, these weights are summed to yield a total LIBRA2 score. Higher scores indicate an unhealthier lifestyle profile and a higher risk of dementia, with a total score ranging from − 6.1 to 25.7. LIBRA2 was constructed in 2024 and represents an update of the original LIBRA index, in which three risk- and protective factors were added (hearing impairment, sleep, social participation). The LIBRA index has been extensively validated to predict brain damage, cognitive decline, incident cognitive impairment and dementia in various population-based studies [28–30] and clinical studies in people with SCD or MCI [31, 32]. LIBRA2 is recently validated in the English Longitudinal Study of Ageing and the Maastricht Ageing Study, showing increased overall performance compared to LIBRA [23]. Yet, validation in clinical samples remains to be established. It has also been included as outcome measures in other multidomain intervention studies targeting the general population [14, 16, 33, 34] and the ongoing 2-year multidomain FINGER-NL trial [35], and has been recommended as a surrogate outcome measure in prevention studies by the World Health Organization’s brain health group [36] and the World Wide FINGER group [20]. Table 2 describes the measurement of each LIBRA2 factor in this study, including the cut-offs that will be applied to assess the presence of the risk factors.

**Table 2:** Assessment of the presence of LIBRA2 risk factors

LIBRA2 factor	Assessment
Obesity	BMI ≥ 25, for persons aged 65 years and older BMI ≥ 28 (risk defined by overweight rather than obesity cut-offs)
Depression	Self-report on depression diagnosis OR PHQ-9 ≥ 10
Hypercholesterolemia	Self-report on hypercholesterolemia diagnosis OR usage of statins OR Total Cholesterol ≥ 5 mmol/L OR LDL cholesterol ≥ 3.5 mmol/L
Heart disease	Self-report on coronary heart disease diagnosis OR using drugs for heart disease (e.g. anticoagulants)
Hypertension	Self-report of hypertension diagnosis OR usage of antihypertensives OR mean diastolic blood pressure ≥ 90 OR mean systolic blood pressure ≥ 140
Diabetes	Self-report on diabetes type 2 diagnosis OR usage of glucose-lowering drugs (e.g. insulin, metformin) OR HbA1c ≥ 48 mmol/mol
Kidney disease	Self-report of chronic kidney disease diagnosis OR eGFR measure of < 60
Hearing impairment	Self-report of hearing impairment OR hearing aids
Sleep	Low sleep quality indicated by the GSKS score ≥ 5
Physical activity	EPIC physical activity questionnaire in inactive or moderately inactive category following the Cambridge physical activity index
Cognitive activity	Low cognitive activity indicated by the Cognitive and Leisure Activities Scale ≤ 30
Social participation	Low social engagement indicated by the 6-item Lubben social network Scale ≤ 14
Smoking	Self-report of current smoking
Alcohol	Self-report quantity of > 7 glasses per week
Healthy diet	No adherence to healthy diet indicated by the MEDAS score *<* 8

*Abbreviations*: *BMI *body mass index, *PHQ-9 *Patient Health Questionnaire-9, *LDL *low-density lipoprotein, *HbA1c *glycated hemoglobin, *eGFR* estimated Glomerular Filtration Rate, *GSKS *Groningen Sleep Quality Scale, *EPIC *European Prospective Investigation into Cancer and Nutrition, *MEDAS *Mediterranean Diet Adherence Screener

### Secondary outcomes

#### Individual LIBRA2 factors

Measures of all individual factors of the LIBRA2 are included as separate secondary outcomes. These include questionnaires on mediterranean diet (Mediterranean Diet Adherence Screener; MEDAS) [37], physical activity (European Prospective Investigation into Cancer and Nutrition; EPIC) [38], cognitive activity (Cognitive and Leisure Activities Scale) [39], depression (PHQ-9) [40] and stress (Perceived Stress Scale) [41], sleep quality (GSKS) [42], social participation (Lubben social network scale) [43] and loneliness (De Jong Gierveld 6-item Scale) [44]. Questionnaires have been validated and used in previous Dutch studies [35, 45]. Participants complete these questionnaires online at home (via Castor EDC). If this is not feasible, participants are provided the opportunity to complete the questionnaires on paper. Smoking and alcohol use (including duration and quantity) and diagnosis of health conditions (hypertension, hypercholesterolemia, coronary heart disease, diabetes, kidney disease, depression, sleep disorders) are assessed through self-report.

Objective measures to define obesity and hypertension include height, weight, and blood pressure (average of three measures while sitting). Lab measures are used to objectively define hypercholesterolemia (through total cholesterol, LDL cholesterol, HDL cholesterol, and triglyceride levels), diabetes (through HbA1c levels), and kidney disease (through creatinine/eGFR levels). These are determined in the blood by standard local protocols (5 ml serum for HbA1c, 4 ml heparin for creatinine and lipids). The presence of health conditions is operationalized through either the presence of a self-reported diagnosis or abnormal values of objective measures, using the same cut-offs as used for calculating LIBRA2 (Table 2).

#### Cognitive functioning

Cognition will be assessed with neuropsychological assessment [46] across different cognitive domains: short- and long-term memory (15-Word Verbal Learning Test) [47], working memory (Wechsler Adult Intelligent Scale digit span) [48], attention (Trail Making Test A), executive functions (Trail Making Test B) [49], information processing speed (Digit Symbol Substitution Test) [50] and language (semantic word fluency, category animals) [51]. At follow-up, parallel tests will be performed for the 15-Word Verbal Learning Test and semantic fluency (category professions). Cognitive testing will be performed according to standard local guidelines of the memory clinic. Tests will be performed and scored by researchers trained in neuropsychological testing and z-scores will be calculated to standardize the scores using local norms. Conversion to MCI or dementia during the study will also be collected if available (based on clinical diagnosis). At follow-up, measures of cognitive functioning are collected by researchers blinded to the intervention group.

#### Daily functioning, psychological and knowledge outcomes

To what extent participants have difficulties in everyday functioning is measured with Amsterdam Instrumental Activities of Daily Living Questionnaire (A-IADL-Q) [52]. Psychological outcomes include locus of control (Health locus of Control Scale) [53], self-efficacy (General Self-Efficacy scale) [54], and mastery (Pearlin Mastery Scale) [55]. In addition, knowledge on dementia risk is assessed (Knowledge on dementia risk questionnaire). Questionnaires have been validated and used in previous Dutch studies [35, 56].

#### Process evaluation

To identify barriers, and facilitators and evaluate strategies for sustainable implementation of the lifestyle intervention in this study, we perform a mixed-method process evaluation. Based on the UK Medical Research Council guidance on process evaluation of complex interventions [57], we collect data on implementation, mechanisms of impact and context. Implementation includes reach (proportion of eligible members of the target group that participated in the intervention), recruitment (sources and procedures for recruitment and reasons for joining or not), fidelity (the extent to which the intervention was delivered as intended), dose delivered (how much of the intervention was provided) and dose received (how much of the delivered intervention was assimilated by participants who were engaged). Mechanisms of impact covers experiences and usefulness of the intervention. Context includes external characteristics of stakeholders and facilitating factors and barriers to delivery of the intervention. Table 3 provides a detailed summary of the process evaluation objectives and methods employed, organized by the domains of implementation, mechanisms of impact and context according to the MRC framework for each stakeholder. Stakeholders in the process evaluation are patients, lifestyle coaches and healthcare professionals (e.g. neurologist, research nurse, general practitioners and community-based lifestyle initiatives). Data will be gathered at different time points throughout the intervention from different stakeholder groups by means of questionnaires, interviews and/or focus groups. Patients will receive three questionnaires throughout one year; lifestyle coaches and other healthcare professionals will receive one questionnaire once randomization is completed. For the post-intervention interviews and/or focus groups with patients, a subset will be selected using purposeful sampling based on sociodemographic factors aiming to include 12–20 participants (3–5 per arm per site) representative of the memory clinic population. All lifestyle coaches and healthcare professionals are invited to participated in a semi-structured interview. Other data registration consists of field notes from conversations, phone calls, meetings, email and measurement observations from our researcher with patients and healthcare professionals (e.g. electronic health record or case report forms and other databases). Also, patients’ usage of BreinZorg is automatically registered. Lifestyle coaches keep pre-structured logs of coach-sessions with the patients. The extent to which lifestyle coaches deliver the intervention according to motivational interviewing guidelines will be assessed using the Motivational Interviewing Treatment Integrity (MITI 4.2.1) scale [58]. We aim to code at least four sessions with different patients of each lifestyle coach (weighed for total number of patients counselled) to provide a reliable competency score for fidelity across the trial.

**Table 3:** Overview of the process evaluation objectives and methods in the different stakeholder groups

Stakeholder Method Timepoint Domain	Patients (both arms)				Lifestyle coach			Healthcare professional		
	Questionnaire			Interview	Questionnaire	Interview	Data registration	Questionnaire	Interview	Data registration
	T = 0	T = 3	T = 12	T = 12^*^	Randomization completed	T = 12	Continuous	Randomization completed	T = 12	Continuous
Implementation										
Reach							field notes			field notes
Recruitment	x			x				x	x	
Fidelity					x	x	audio recording, field notes	x	x	field notes
Dose delivered					x	x	pre-structured logs			
Dose received		x	x	x			field notes, BreinZorg	x	x	field notes
Mechanisms of impact										
Experiences and usefulness		x	x	x	x	x		x	x	
Context										
Characteristics					x			x		
Facilitators and barriers			x	x	x	x		x	x	

Domains are based on UK Medical Research Council guidance on process evaluation of complex interventions. Field notes are documentations in electronic health record or case report forms and/or other databases on conversations, phone calls, meetings, email and measurement observations with patients and healthcare professionals

*Subset *N* = 12–20 (3–5 participants per arm per site)

#### Cost-effectiveness

In an early health-technology assessment, the cost-effectiveness of the lifestyle intervention will be estimated from a societal perspective (reflecting medical sector, social sector, patient and family, and productivity loss) over a lifetime horizon. Measures involve health related quality of life (EuroQol-5D-5 L) [59], capability (ICEpop CAPability measure for Older people, ICECAP-O) [60]. In addition, questionnaires for care use (Medical Consumption Questionnaire) [61] and productivity (Productivity Costs Questionnaire) [62] are performed.

#### Adherence

Additionally, adherence measures will be recorded to track real-world feasibility and implementation. Adherence measures include the number of appointments with the lifestyle coach or research visits missed, the number of personal goals set and reached, the number of vouchers provided and their usage, and a usage log of BreinZorg (login times, completion of learning modules).

#### Other measures

Demographical data (age, sex, education, household income, work, ethnicity, marital status, living condition) will be collected. Participants are additionally asked whether they are already following any lifestyle-related treatment, for example by a dietician or cardiovascular management at the general practitioner. As part of the lab measures, additional blood is drawn (6 ml) and stored in the biobank for determining APOE genotype (if not already available) and biomarkers (e.g. amyloid and p-tau) in the future.

### Sample size

For power calculation, we used mean scores of the primary outcome, the LIBRA2 score, in people with SCD and MCI as reported in a previous study [31]. A 1-point difference in LIBRA2 scores between the intervention and control group at the end of the study would translate into a Cohen’s d = 0.35. To detect such a small to moderate effect size with 80% power in two-sided testing at an alpha-level of 0.05 and a 1:1 treatment allocation ratio, 133 patients per group are needed. Further assuming a 10% dropout rate during the trial means that we need 148 participants per group, resulting in a total sample size of 296, which was rounded up to 300.

### Data analysis

Demographics will be described and compared between centers and diagnostic status by using t-tests (continuous data) and chi-squared tests (categorical data). Assumptions will be checked, and transformations will be used where necessary.

#### Effectiveness analysis

We will use linear mixed model analyses to test the effect of the intervention on the primary and secondary outcome variables (except secondary measures collected for process evaluation and cost-effectiveness) as these models generally handle missing outcome data well and thereby align with the intention-to-treat principle, even if only baseline values are available [63]. Models will use a random intercept (individuals) and, if likelihood ratio testing suggests better fit, random slopes (time). Treatment group (intervention or control) and time (baseline and 1-year follow-up) will be included as fixed effects, and study site and diagnostic group (SCD or MCI) as fixed covariates. Additional covariates will be added if randomization was not successful in balancing other characteristics (e.g. age, gender and education) between treatment groups. An interaction term between treatment group and time will be included as fixed effect. The intention-to-treat principle is used by including all randomized participants in the analyses, including dropouts and non-adherent participants. Missing primary and secondary outcome data are considered as missing-at-random (i.e., missingness is probabilistic and conditional on the covariates included in the analyses) and handled efficiently through maximum likelihood estimation of expected scores in the linear mixed model [64]. We will additionally assess differences in effectiveness and adherence between the SCD and MCI groups using stratified analysis. All analyses will be two-sided at an alpha level of 0.05 and conducted in Stata 18 or higher. A per-protocol sensitivity analysis will be performed to assess whether results remain consistent when excluding participants with low adherence to the intervention.

#### Process evaluation analysis

For the quantitative data, descriptive statistics (mean, SD, proportions) will be used to report on patients’, healthcare professionals’ and lifestyle coaches’ characteristics, as well as the results of the closed-ended questions and pre-structured logs of lifestyle coach sessions. Qualitative data (open-ended questions, interviews and focus-groups) will be analyzed using thematic content analysis to identify barriers and facilitators for adoption, implementation and continuation of the intervention program. Accordingly, all interviews and focus groups will be audiotaped, fully transcribed verbatim and anonymized. Field notes will be reviewed for additional information related to the identified themes.

#### Cost-effectiveness analysis

For cost-effectiveness analyses, a previous developed decision-analytic model framework for primary prevention intervention will be used [65, 66]. This Markov-type model will simulate the trial starting population of SCD and MCI and the onset of dementia based on the LIBRA2 modifiable lifestyle risk factors and the dementia onset rate and progression in a memory clinic setting. It estimates the person-years living in SCD, MCI and dementia (severity) stages, and associated quality-adjusted life years (QALY) based on EuroQol-5D-5 L and ICECAP-O and costs from a societal perspective for the Dutch setting, including care use (Medical Consumption Questionnaire) [61] and productivity (Productivity Costs Questionnaire) [62] multiplied with unit prices.

Uncertainty will be addressed using deterministic and probabilistic sensitivity analyses. Heterogeneity in terms of intervention response specific to (demographic and socio-economic) characteristics will be assessed dependent on the trial results. At last, a budget impact analysis will be performed using inputs among which incidence, uptake/market penetration, labour requirement, potential “catch-up” population. These inputs will be obtained from the study results regarding effectiveness of the intervention and process evaluation, as well as literature. The Dutch guideline for economic evaluation in healthcare will be followed for the cost-effectiveness analysis and budget impact analysis. Results will be published open-access following the CHEERS reporting checklist [67], and the model will be made available open-source.

## Discussion

This design paper provides an overview of the rationale and study design of LIGHT, a 12-month multicenter RCT investigating the effects of a personalized multidomain lifestyle intervention to improve brain health in memory clinic patients. This study will show if a lifestyle intervention including three counselling sessions with a lifestyle coach, a voucher program and an online self-management platform is (cost-)effective and possible to implement in Dutch hospitals.

Translating intentions into actual and sustainable health behavior on the long-term is challenging and involves many individual barriers, such as socio-economic status and access to (affordable) resources, social support, and health literacy [68, 69]. To increase involvement in the intervention, participants will be individually guided by a coach across three sessions [70]. The use of motivational interviewing helps participants to articulate their own desires and their underlying reasons. This has been shown to be effective in the motivation to change behaviors and increase adherence to lifestyle intervention programs [71]. By implementing a personalized intervention, in which participants formulate their own targets and goals to fit their individual needs together with a coach, we aim to increase internal motivation of participants to successfully change their behavior and maintain this newly adopted behavior in the long term.

Besides personalized coaching and goal setting, a new and unique component in our personalized intervention is a voucher program, connecting with regional partners to promote brain-healthy behavior. Vouchers might help participants initiating their aimed goals by giving them free or discounted access to facilities such as sports group sessions, fresh vegetables and fruit from local farms, a subscription for unlimited access to museums, dance and arts courses, mobile phone applications for making new social contacts, and more. The goal is to lower the bar to try out new brain-healthy activities. Persons with lower socio-economic status usually carry higher risk and have more difficulties maintaining healthy lifestyles [72]. Particularly in this target group, the voucher program can financially support and stimulate a brain healthy lifestyle, and by this, increase health equity. Vouchers are specifically targeted at domains that have room for improvement (based on the LIBRA2). The idea is based on the successful implementation of the nationwide voucher program in Luxembourg, as part of the Programme Dementia Prevention (PDP) [27]. Evaluation as part of this study will explore its effectiveness and potential for wider implementation.

In addition, the use of Breinzorg.nl completes the intervention by giving access to a structured and reliable platform with all relevant information regarding risk reduction for dementia. It is developed as a low-cost and effective tool to create awareness and knowledge on dementia reduction.

Recently, in the Netherlands, several hospitals are establishing lifestyle front offices for integrating lifestyle medicine in the routine treatment of patients in collaboration with community-based lifestyle initiatives [70, 73]. If the LIGHT intervention shows to be (cost-)effective, such lifestyle front offices might serve as an opportunity to implement the intervention across hospitals in the Netherlands. This approach aligns with the idea of installing dedicated brain health services, which has emerged more recently in the field [74, 75].

As we are working towards implementation in regular healthcare, we will not only assess clinical effectiveness of the intervention but also explore qualitative analyses for process evaluation and cost-effectiveness [76]. These analyses are crucial for the effective implementation of the intervention in the memory clinic and will help finetuning and improving the intervention where necessary. This is essential to align the lifestyle intervention properly with the needs of participants, lifestyle coaches, as well as healthcare professionals in a way that it is realistic and feasible.

## Conclusions

Results from LIGHT will provide insights in the effectiveness of a newly developed personalized multidomain lifestyle intervention in Dutch memory clinics. Besides clinical effectiveness, we will also explore the implementation in the healthcare setting based on the barriers and facilitating factors, as well as cost-effectiveness. Ultimately, the goal is to implement and provide access to an effective intervention to all memory clinic patients that might benefit from lifestyle changes to reduce dementia risk. Findings may inspire other countries to develop and implement similar lifestyle interventions within the hospital setting in the form of lifestyle front offices that memory clinic patients can be referred to.

## Supplementary Information

Supplementary Material 1.

## Data Availability

No datasets were generated or analysed during the current study.
