# Supplementary material for: The Lifestyle Intervention in memory clinics of General and academic Hospitals Trial (LIGHT): Rationale and study design of a randomized controlled trial to reduce modifiable dementia risk
Source: Alzheimers Res Ther. 2026 Jan 8;18:31. doi: 10.1186/s13195-025-01947-9 (PMC12882484; doi:10.1186/s13195-025-01947-9)
Supplement: Supplementary file 1 — Supplementary Material 1. [file 13195_2025_1947_MOESM1_ESM.docx]

**Supplementary Table 1. Weights of the factors included in the LIBRA2 index.**

| **Factor** | **Relative risk** | **Weight LIBRA2^a^** |
| --- | --- | --- |
| High alcohol intake | 1.18 [1] | +1.0 |
| Coronary heart disease | 1.55 [2] | +2.6 |
| High physical activity | 0.73 [3] | -1.9 |
| Chronic kidney disease | 1.35 [4] | +1.8 |
| Diabetes | 1.43 [5] | +2.2 |
| Hypercholesterolemia | 1.54 [6] | +2.6 |
| Smoking | 1.52 [7] | +2.5 |
| Obesity | 1.45 [8] | +2.2 |
| Hypertension | 1.20 [9] | +1.1 |
| Healthy diet | 0.82 [10] | -1.2 |
| Depression | 1.98 [11] | +4.1 |
| High cognitive activity | 0.61 [12] | -3.0 |
| Hearing impairment | 1.49 [13] | +2.4 |
| Low social participation | 1.41 [14] | +2.1 |
| Sleep disturbances | 1.19 [15] | +1.1 |

Table extracted from Rosenau et al., 2024, see reference for comparison to original LIBRA index [16]. Abbreviations: LIBRA2, updated LIfestyle for BRAin health.

^a^The weights for the factors in LIBRA2 were calculated by taking the natural logarithms of their new relative risks. Subsequently those were standardized by taking the lowest absolute value of the natural logarithms as the reference value and dividing each natural logarithm by this value.

**References**

1. Livingston G, Huntley J, Sommerlad A, et al. Dementia prevention, intervention, and care: 2020 report of the Lancet Commission. *Lancet*. Aug 8 2020;396(10248):413-446. doi:10.1016/S0140-6736(20)30367-6

2. Deckers K, Schievink SHJ, Rodriquez MMF, et al. Coronary heart disease and risk for cognitive impairment or dementia: Systematic review and meta-analysis. *PLoS One*. 2017;12(9):e0184244. doi:10.1371/journal.pone.0184244

3. Xu W, Wang HF, Wan Y, Tan CC, Yu JT, Tan L. Leisure time physical activity and dementia risk: a dose-response meta-analysis of prospective studies. *BMJ Open*. Oct 22 2017;7(10):e014706. doi:10.1136/bmjopen-2016-014706

4. Deckers K, Camerino I, van Boxtel MP, et al. Dementia risk in renal dysfunction: A systematic review and meta-analysis of prospective studies. *Neurology*. Jan 10 2017;88(2):198-208. doi:10.1212/WNL.0000000000003482

5. Xue M, Xu W, Ou YN, et al. Diabetes mellitus and risks of cognitive impairment and dementia: A systematic review and meta-analysis of 144 prospective studies. *Ageing Res Rev*. Nov 2019;55:100944. doi:10.1016/j.arr.2019.100944

6. Anstey KJ, Cherbuin N, Herath PM. Development of a new method for assessing global risk of Alzheimer's disease for use in population health approaches to prevention. *Prev Sci*. Aug 2013;14(4):411-21. doi:10.1007/s11121-012-0313-2

7. Niu H, Qu Y, Li Z, et al. Smoking and Risk for Alzheimer Disease: A Meta-Analysis Based on Both Case-Control and Cohort Study. *J Nerv Ment Dis*. Sep 2018;206(9):680-685. doi:10.1097/NMD.0000000000000859

8. Qu Y, Hu HY, Ou YN, et al. Association of body mass index with risk of cognitive impairment and dementia: A systematic review and meta-analysis of prospective studies. *Neurosci Biobehav Rev*. Aug 2020;115:189-198. doi:10.1016/j.neubiorev.2020.05.012

9. Ou YN, Tan CC, Shen XN, et al. Blood Pressure and Risks of Cognitive Impairment and Dementia: A Systematic Review and Meta-Analysis of 209 Prospective Studies. *Hypertension*. Jul 2020;76(1):217-225. doi:10.1161/HYPERTENSIONAHA.120.14993

10. Liu YH, Gao X, Na M, Kris-Etherton PM, Mitchell DC, Jensen GL. Dietary Pattern, Diet Quality, and Dementia: A Systematic Review and Meta-Analysis of Prospective Cohort Studies. *J Alzheimers Dis*. 2020;78(1):151-168. doi:10.3233/JAD-200499

11. Cherbuin N, Kim S, Anstey KJ. Dementia risk estimates associated with measures of depression: a systematic review and meta-analysis. *BMJ Open*. Dec 21 2015;5(12):e008853. doi:10.1136/bmjopen-2015-008853

12. Yates LA, Ziser S, Spector A, Orrell M. Cognitive leisure activities and future risk of cognitive impairment and dementia: systematic review and meta-analysis. *Int Psychogeriatr*. Nov 2016;28(11):1791-1806. doi:10.1017/S1041610216001137

13. Ford AH, Hankey GJ, Yeap BB, Golledge J, Flicker L, Almeida OP. Hearing loss and the risk of dementia in later life. *Maturitas*. Jun 2018;112:1-11. doi:10.1016/j.maturitas.2018.03.004

14. Kuiper JS, Zuidersma M, Oude Voshaar RC, et al. Social relationships and risk of dementia: A systematic review and meta-analysis of longitudinal cohort studies. *Ageing Res Rev*. Jul 2015;22:39-57. doi:10.1016/j.arr.2015.04.006

15. Shi L, Chen SJ, Ma MY, et al. Sleep disturbances increase the risk of dementia: A systematic review and meta-analysis. *Sleep Med Rev*. Aug 2018;40:4-16. doi:10.1016/j.smrv.2017.06.010

16. Rosenau C, Köhler S, van Boxtel M, Tange H, Deckers K. Validation of the Updated “LIfestyle for BRAin health”(LIBRA) Index in the English Longitudinal Study of Ageing and Maastricht Aging Study. Journal of Alzheimer’s Disease. 2024;101(4):1237-48.
